# The bacterial metabolite, lithocholic acid, has antineoplastic effects in pancreatic adenocarcinoma

**DOI:** 10.1038/s41420-024-02023-1

**Published:** 2024-05-23

**Authors:** Szandra Schwarcz, Patrik Kovács, Petra Nyerges, Gyula Ujlaki, Adrienn Sipos, Karen Uray, Péter Bai, Edit Mikó

**Affiliations:** 1 Department of Medical Chemistry, Faculty of Medicine, University of Debrecen, Debrecen, 4032 Hungary; 2 HUN-REN-UD Cell Biology and Signaling Research Group, Debrecen, 4032 Hungary; 3 MTA-DE Lendület Laboratory of Cellular Metabolism, Debrecen, 4032 Hungary; 4 Research Center for Molecular Medicine, Faculty of Medicine, University of Debrecen, Debrecen, 4032 Hungary

**Keywords:** Cancer metabolism, Cancer stem cells, Pancreatic cancer

## Abstract

Lithocholic acid (LCA) is a secondary bile acid. LCA enters the circulation after bacterial synthesis in the gastrointestinal tract, reaches distantly located cancer cells, and influences their behavior. LCA was considered carcinogenic, but recent studies demonstrated that LCA has antitumor effects. We assessed the possible role of LCA in pancreatic adenocarcinoma. At the serum reference concentration, LCA induced a multi-pronged antineoplastic program in pancreatic adenocarcinoma cells. LCA inhibited cancer cell proliferation and induced mesenchymal-to-epithelial (MET) transition that reduced cell invasion capacity. LCA induced oxidative/nitrosative stress by decreasing the expression of nuclear factor, erythroid 2-like 2 (NRF2) and inducing inducible nitric oxide synthase (iNOS). The oxidative/nitrosative stress increased protein nitration and lipid peroxidation. Suppression of oxidative stress by glutathione (GSH) or pegylated catalase (pegCAT) blunted LCA-induced MET. Antioxidant genes were overexpressed in pancreatic adenocarcinoma and decreased antioxidant levels correlated with better survival of pancreatic adenocarcinoma patients. Furthermore, LCA treatment decreased the proportions of cancer stem cells. Finally, LCA induced total and ATP-linked mitochondrial oxidation and fatty acid oxidation. LCA exerted effects through the farnesoid X receptor (FXR), vitamin D receptor (VDR), and constitutive androstane receptor (CAR). LCA did not interfere with cytostatic agents used in the chemotherapy of pancreatic adenocarcinoma. Taken together, LCA is a non-toxic compound and has antineoplastic effects in pancreatic adenocarcinoma.

## Introduction

Pancreatic adenocarcinoma is the seventh leading cause of cancer deaths worldwide, with 496,000 new cases and 466,000 deaths in 2020 [1]. Patients are typically diagnosed at an advanced stage because early diagnostic markers are lacking and patients are asymptomatic during the early stage of the disease. Furthermore, the high metastatic potential of the disease and resistance to chemotherapy results in a poor prognosis [2]. Therefore, a better understanding of the pathogenesis of pancreatic adenocarcinoma is essential.

The compositions of multiple microbiome compartments change in neoplastic diseases. These changes are termed oncobiosis, and the resulting bacterial community is the oncobiome [3–5]. Oncobiosis itself is unlikely to induce tumors but can promote tumor growth and metastases [3, 6]. The oncobiome supports a subset of cancer hallmarks, including invasion and metastasis, angiogenesis and inflammation, deregulation of cellular metabolism, and avoidance of immune destruction [7–10].

The anatomical location of the pancreas close to the gastrointestinal tract supports bidirectional communication between the gut and the pancreas, which is often called the gut-pancreas, or gut-pancreas-liver axis. The composition of oral [11] and duodenal [12] microbiota is altered in pancreatic adenocarcinoma. Oral and intestinal microbes can translocate and colonize the pancreatic duct and the pancreas [12–14]. Interestingly, fungal colonization is also associated with pancreatic adenocarcinoma [15]. Bacteria colonizing the pancreas promote inflammation and support tumorigenesis and progression [13, 16]. Invasive bacteria may also subsequently enter the circulation. Invasive bacteria are often strongly immunogenic due to LPS production that can activate Toll-like receptors (TLRs) and induce the production of proinflammatory cytokines (e.g. CXCLs, IL-6) through NF-kB [17].

In addition to the direct immunogenicity of the microbiome, an endocrine-like function of the microbiome has also been described in several cancers [18–20] including pancreatic adenocarcinoma. Due to its size, the intestinal microbiome has considerable biosynthetic capacity and can synthesize bioactive metabolites, which can enter the circulation and stimulate hormone-like effects on distant targets, such as tumor cells.

In our previous studies, we identified several cytostatic bacterial metabolites from the intestinal microbiome in breast cancer. In terms of their chemical structure, these substances are very heterogeneous [7–9, 21–23]. The role of bacterial metabolites in the context of pancreatic adenocarcinoma is poorly understood but several metabolites have been identified. These bacterial metabolites can elicit pro- [24, 25] and anticarcinogenic [26, 27] effects in pancreatic adenocarcinoma.

Primary bile acids (BAs), cholic acid (CA), and chenodeoxycholic acid (CDCA) synthesized from cholesterol in the liver and conjugated to glycine or taurine, are secreted into the duodenum [28]. BAs are reabsorbed in the ileum and returned to the liver through the portal vein or converted to secondary bile acids, including deoxycholic acid (DCA), lithocholic acid (LCA), and ursodeoxycholic acid (UDCA), in the colon [29].

The gut microbiome contributes to the conversion of primary BAs to secondary BAs. Bile acid hydrolases (BSH) in intestinal bacteria deconjugate primary BAs by hydrolyzing glycine and taurine conjugates. BSH is broadly expressed in *Bacteroides, Bifidobacterium*, *Clostridium, Lactobacillus, Listeria*, and *Enterococcus*. Another critical transformation step is catalyzed by 7 α/β dehydroxylase found mainly in *Clostridia* and *Eubacteria* [30, 31]. LCA is formed from chenodeoxycholic acid (CDCA) via deconjugation of CDCA conjugates and dehydroxylation of carbon 7 by 7 α/β hydroxysteroid dehydrogenase (7-HSDH; a key enzyme in LCA generation) [29]. The enzymes mediating 7-dehydroxylation of bile acids are organized into the bile acid-inducible (*bai*) operon; the *baiH* ORF codes for 7-HSDH in most bacterial species [29].

The interaction between the gut microbiome and BAs is bidirectional. The gut microbiota regulate bile acid metabolism, including the synthesis, conjugation, and conversion of primary to secondary bile acids [32]. Conversely, BAs have the potential to change the composition of the microbiome [33, 34] and facilitate bacterial translocation [35], a key step in the carcinogenesis of pancreatic adenocarcinoma. The sensitivity of bacteria to BA varies. For example, *Enterococci* are BA-resistant, which may allow *Enterococci* to accumulate in pancreatic adenocarcinoma [36].

Originally, LCA was considered procarcinogenic [37–39]; however, recent studies showed that LCA also has anti-tumor effects. LCA has antineoplastic effects in a variety of tumors, including neuroblastoma [40], prostate cancer [41, 42], nephroblastoma [43], gallbladder cancer [44], liver cancer [45], and breast cancer [7, 9, 46]. Due to the widespread antineoplastic effects of LCA, we investigated its effects in pancreatic adenocarcinoma.

## Results

### LCA inhibits the proliferation of pancreatic adenocarcinoma cells

**Fig. 1:**
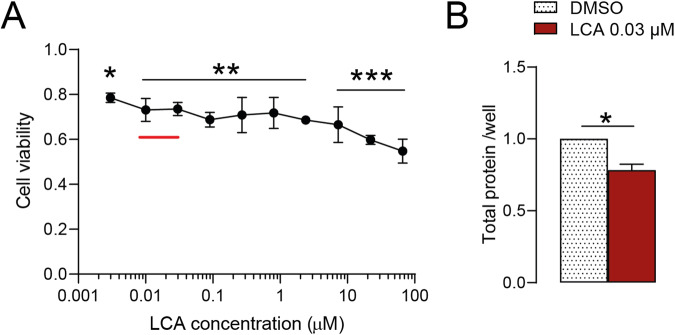
LCA treatment decreases the proliferation of pancreatic adenocarcinoma cells. Capan-2 cells were treated with LCA at the indicated concentrations for 48 h. **A** Cell viability was measured with the MTT assay (*n* = 3) and **B** total protein concentrations were measured using the SRB assay (*n* = 3). Data are shown as means ± SEM. Statistical differences in cell viability were determined by one-way ANOVA and Dunnett post hoc test, where LCA-treated samples were compared to the DMSO control sample. Protein concentrations in LCA-treated and DMSO control samples were compared with paired *t* tests. *, **, and *** indicate *p* < 0.05, *p* < 0.01, and *p* < 0.001, respectively, between DMSO and LCA-treated groups. The red line indicates the normal serum reference concentration range (0.01–0.03 µM). DMSO dimethyl sulfoxide, LCA lithocholic acid.

We investigated whether LCA could influence Capan-2 cell proliferation. Cell numbers were determined using 3-(4,5-dimethylthiazol-2-yl)-2,5-diphenyltetrazolium bromide (MTT) assays after treating cells with increasing concentrations of LCA (0.003 µM–66 µM). LCA from 0.01 µM concentration upwards, which encompasses the serum reference concentration of LCA (0.01–0.03 µM), significantly inhibited the proliferation of Capan-2 cells [7, 47–49] (Fig. 1A). Similar to the MTT assay, 0.03 µM LCA slowed the proliferation of Capan-2 cells measured using the sulforhodamine B (SRB) assay (Fig. 1B). Importantly, LCA at the same concentration range (0.01 µM–10 µM) did not affect the proliferation of non-transformed, primary human skin fibroblasts [7], suggesting that the effects of LCA are tumor cell-specific.

### LCA decreases epithelial-mesenchymal transition (EMT)-related gene expression and cell invasion in pancreatic adenocarcinoma cells

**Fig. 2:**
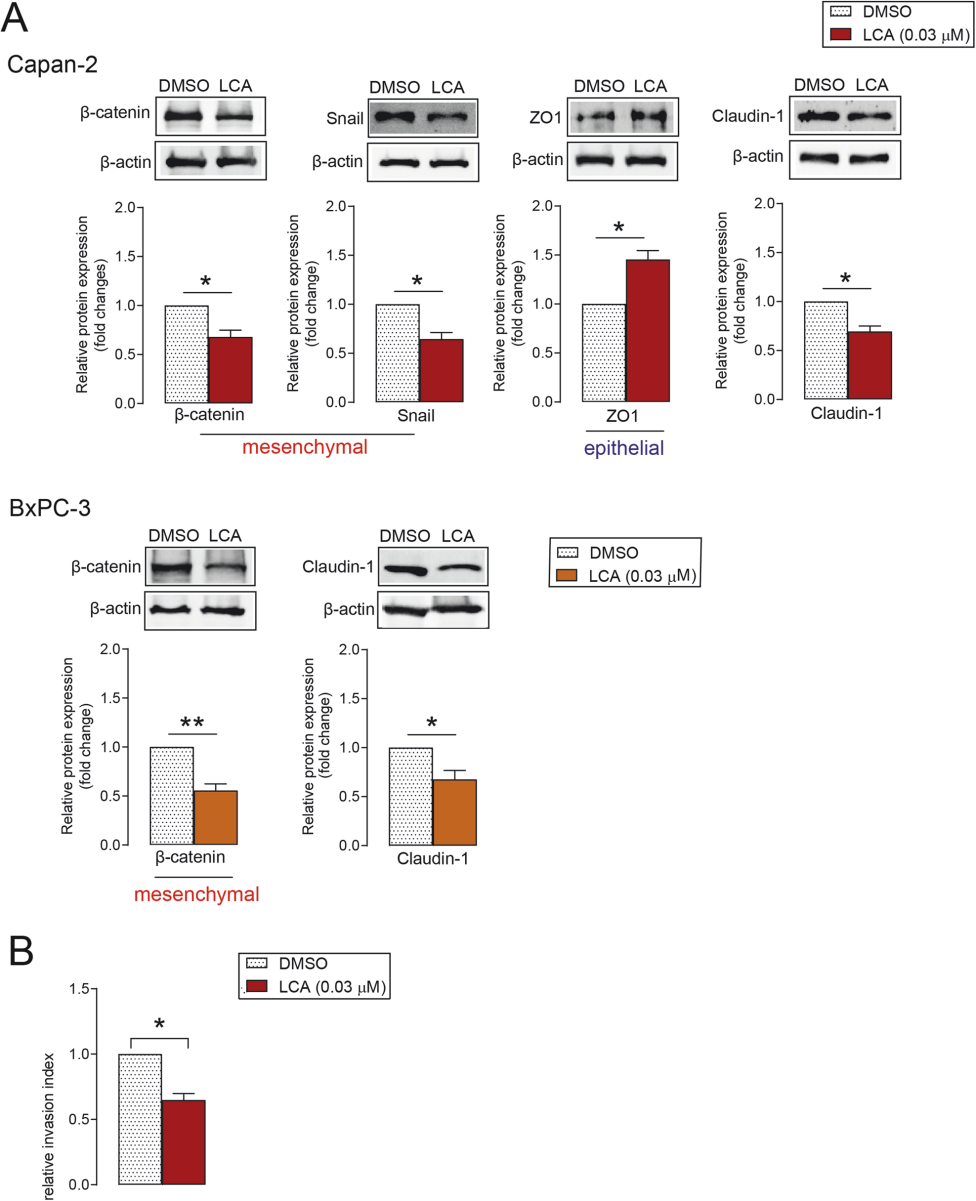
LCA decreases EMT marker expression and the invasion ability of pancreatic adenocarcinoma cells. **A** Capan-2 and BxPC-3 cells were treated with LCA (0.03 µM) or DMSO for 48 h, and EMT marker (mesenchymal: β-catenin, Snail; epithelial: ZO1) and Claudin-1 protein expression levels were determined (*n* = 3, upper panels: representative western blots, lower panels: densitometric analyses of western blots). **B** Cell invasion was measured after treating Capan-2 cells with 0.03 µM LCA for 48 h. The invasion index was calculated (*n* = 3). Means ± SEM are shown. * and **; *p* < 0.05 and *p* < 0.01, respectively, DMSO-treated vs. LCA-treated groups. DMSO dimethyl sulfoxide, LCA lithocholic acid, ZO1 zona occludens 1.

LCA significantly reduced the protein levels of mesenchymal markers, including Snail in Capan-2 cells and β-catenin in Capan-2 and BxPC-3 cells. Expression of the epithelial marker, zonula occludens (ZO1) increased in Capan-2 cells after LCA treatment (Fig. 2A). Interestingly, LCA decreased the tight junction protein, Claudin-1, expression. In line with these results, cell invasion decreased significantly in LCA-treated cells compared with DMSO-treated cells (Fig. 2B). Taken together, LCA inhibits EMT by reducing the expression of EMT-related genes and Claudin-1 to reduce the invasion capacity of pancreatic adenocarcinoma cells.

### High antioxidant expression level in pancreatic adenocarcinoma patients predicts worse clinical outcomes

**Fig. 3:**
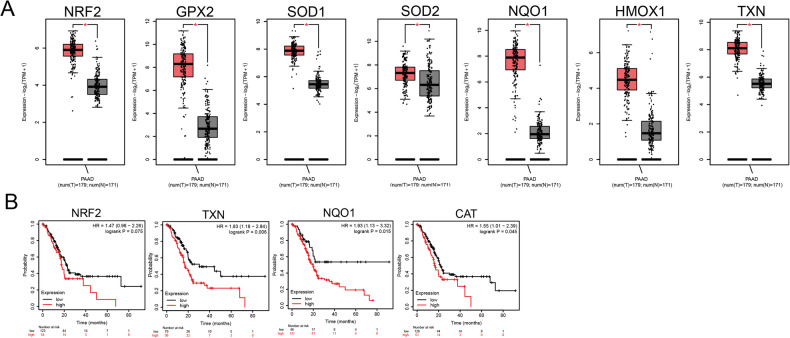
Pancreatic adenocarcinoma tumors overexpress antioxidant genes, and decreased antioxidant levels correlate with better survival of pancreatic adenocarcinoma patients. **A** Boxplot comparing the expression of several antioxidant genes and NRF2 targets in pancreatic adenocarcinoma compared to normal tissue. The expression levels are shown on a log2 (TPM + 1) scale. Images were taken from the Gene Expression Profiling Interactive Analysis online database (http://gepia.cancer-pku.cn) on May 11, 2023. The red boxes represent tumor samples, and the gray boxes represent normal adjacent tissues. **B** Kaplan–Meier curves for overall survival using the Kaplan–Meier Plotter database (www.kmplot.com). The database was accessed on January 30, 2023. The red and black lines indicate patients with higher and lower expressions, respectively. *p < 0.05 tumor vs. normal samples. CAT catalase, GPX2 glutathione peroxidase 2, HMOX1 heme oxygenase 1, NQO1 NAD(P)H quinone dehydrogenase 1, NRF2 nuclear factor erythroid 2-like 2, SOD1,2 superoxide dismutase 1,2, PAAD pancreatic adenocarcinoma, TXN thioredoxin.

We explored the expression of several antioxidants (NRF2, GPX2, SOD1,2) and NRF2 target genes (NQO1, HMOX1, TXN) in 179 tumors and 171 normal tissues from the TCGA/GTEx pancreatic adenocarcinoma (PAAD) dataset. Interestingly, the expression of antioxidants and NRF2 target genes increased in PAAD compared to normal tissue (Fig. 3A). Furthermore, high antioxidant expression was associated with worse overall survival (Fig. 3B). These results indicate that high antioxidant expression is a hallmark of pancreatic adenocarcinoma and may worsen disease outcomes.

### LCA-induced oxidative/nitrosative stress contributes to the reduced expression of EMT-related genes in pancreatic adenocarcinoma cells

**Fig. 4:**
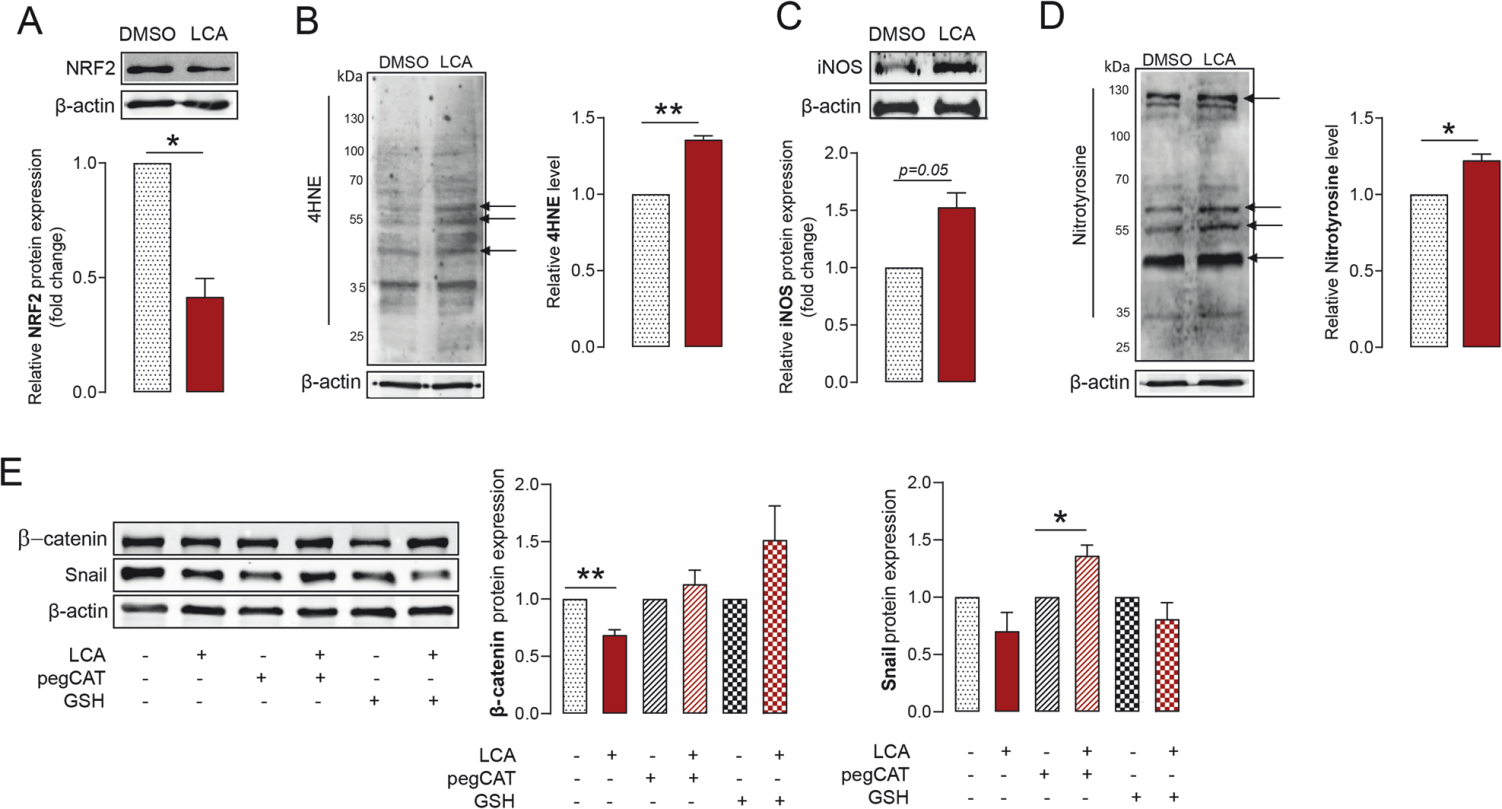
LCA-induced oxidative/nitrosative stress reduces EMT marker expression in pancreatic adenocarcinoma cells. Capan-2 cells were treated with LCA (0.03 µM) for 48 h, and protein levels of **A** NRF2, **B** 4HNE, **C** iNOS, and **D** nitrotyrosine were assessed (*n* = 3, upper and left panels are representative western blots, lower and right panels show the densitometric analyses from independent experiments). (**E**) β-catenin and Snail protein levels were measured in Capan-2 cells after treatment with LCA (0.03 µM) and/or GSH and pegCAT for 48 h (*n* = 3, left panel shows representative western blots and the right panel shows densitometric analyses from the independent experiments). Arrows indicate bands used for the densitometric analysis in the independent experiments. Data are presented as means ± SEM. * and ** indicate *p* < 0.05 and *p* < 0.01, DMSO vs. LCA treated groups. 4HNE 4-hydroxynonenal, DMSO dimethyl sulfoxide, GSH glutathione, iNOS inducible nitric oxide synthase, LCA lithocholic acid, NRF2 nuclear factor, erythroid-derived 2-like 2, pegCAT pegylated catalase.

We showed that antioxidants are overexpressed in human pancreatic adenocarcinoma tissue compared with normal tissue, and overexpression correlates with poor survival. In a previous study on breast carcinoma [9], we demonstrated that breast tumors are protected from reactive species, and LCA-elicits cytostasis by inducing oxidative stress. Here, we investigated whether a similar scenario occurs in pancreatic adenocarcinoma cells. The expression of NRF2, a central regulator of cellular antioxidant defense, decreased in Capan-2 pancreatic adenocarcinoma cells after LCA treatment (Fig. 4A), similar to breast cancer cells. The levels of 4-hydroxynonenal (4HNE)-protein adducts increased after LCA treatment, indicating enhanced lipid peroxidation (Fig. 4B). Furthermore, LCA treatment increased the expression of inducible nitric oxide synthase (iNOS) protein (Fig. 4C), which may lead to increased nitrosative stress in cells and, subsequently, peroxynitrite (ONOO^-^) formation. Nitrotyrosine levels increased in LCA-treated cells (Fig. 4D).

Of note, the application of antioxidants, such as the thiol reductant glutathione (GSH) and pegylated catalase (pegCAT), prevented the decreased expression of β-catenin and Snail induced by LCA. Thus, LCA-induced oxidative/nitrosative stress is important in blocking EMT in pancreatic adenocarcinoma cells (Fig. 4E).

### LCA reduces the expression of cancer stem cell markers in pancreatic adenocarcinoma cells

**Fig. 5:**
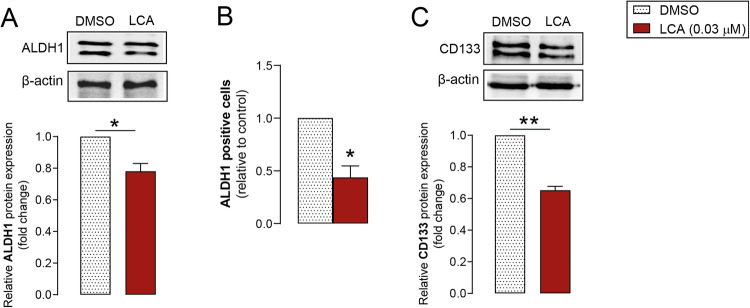
LCA reduces ALDH1 and CD133 protein levels in pancreatic adenocarcinoma cells. Capan-2 cells were treated with LCA (0.03 µM) or DMSO for 48 h. **A** ALDH1 protein levels measured by Western blotting (*n* = 3). **B** ALDH-positive cells detected by FACS analysis (*n* = 3 in triplicates). **C** CD133 protein expression determined by Western blotting (*n* = 3). Data are presented as means ± SEM. *p < 0.05, **p < 0.01, DMSO vs. LCA-treated groups. ALDH1 aldehyde dehydrogenase 1, DMSO dimethyl sulfoxide, LCA lithocholic acid.

The effects of LCA on the proportions of cancer stem cells in cultured cells were assessed using aldehyde dehydrogenase 1 (ALDH1) and CD133 markers. LCA significantly decreased ALDH1 protein levels (Fig. 5A) and reduced ALDH-positive Capan-2 cells measured using the Aldefluor assay (Fig. 5B). Consistent with these results, the protein expression of CD133 decreased after LCA treatment (Fig. 5C). These results demonstrate that LCA reduced the proportion of cancer stem cells among pancreatic adenocarcinoma cells.

### LCA induces mitochondrial oxidative phosphorylation in pancreatic adenocarcinoma cells

**Fig. 6:**
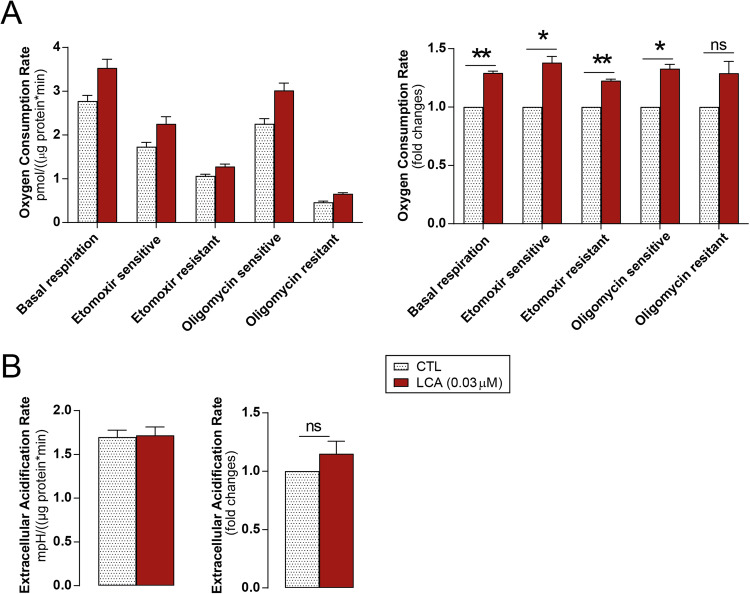
LCA increases mitochondrial respiration in pancreatic adenocarcinoma cells. Mitochondrial oxygen consumption and glycolysis were measured in Capan-2 cells treated with LCA (0.03 µM) or DMSO for 48 h using a Seahorse XF96 analyzer. OCR (**A**) and ECAR (**B**) were calculated (*n* = 3). Parts A and B show representative experiments and fold change values from three independent experiments. * and **p < 0.05 and *p* < 0.01, DMSO vs. LCA-treatment. DMSO dimethyl sulfoxide, ECAR extracellular acidification rate, LCA lithocholic acid, ns non-significant, OCR oxygen consumption rate.

Cellular metabolism was assessed in LCA-treated Capan-2 cells. LCA significantly enhanced basal respiration, etomoxir-sensitive respiration (fatty acid oxidation), etomoxir-resistant respiration (glucose and amino acid oxidation), and oligomycin-sensitive, ATP-linked respiration (Fig. 6A). LCA treatment did not significantly influence the oligomycin-resistant fraction of mitochondrial oxidation, corresponding to uncoupled respiration (Fig. 6A). Furthermore, LCA did not affect glycolysis (ECAR) in Capan-2 cells. (Fig. 6B). These results highlight a key role of LCA in mitochondrial oxidation.

### LCA-induced effects are mediated by FXR, CAR, and VDR

**Fig. 7:**
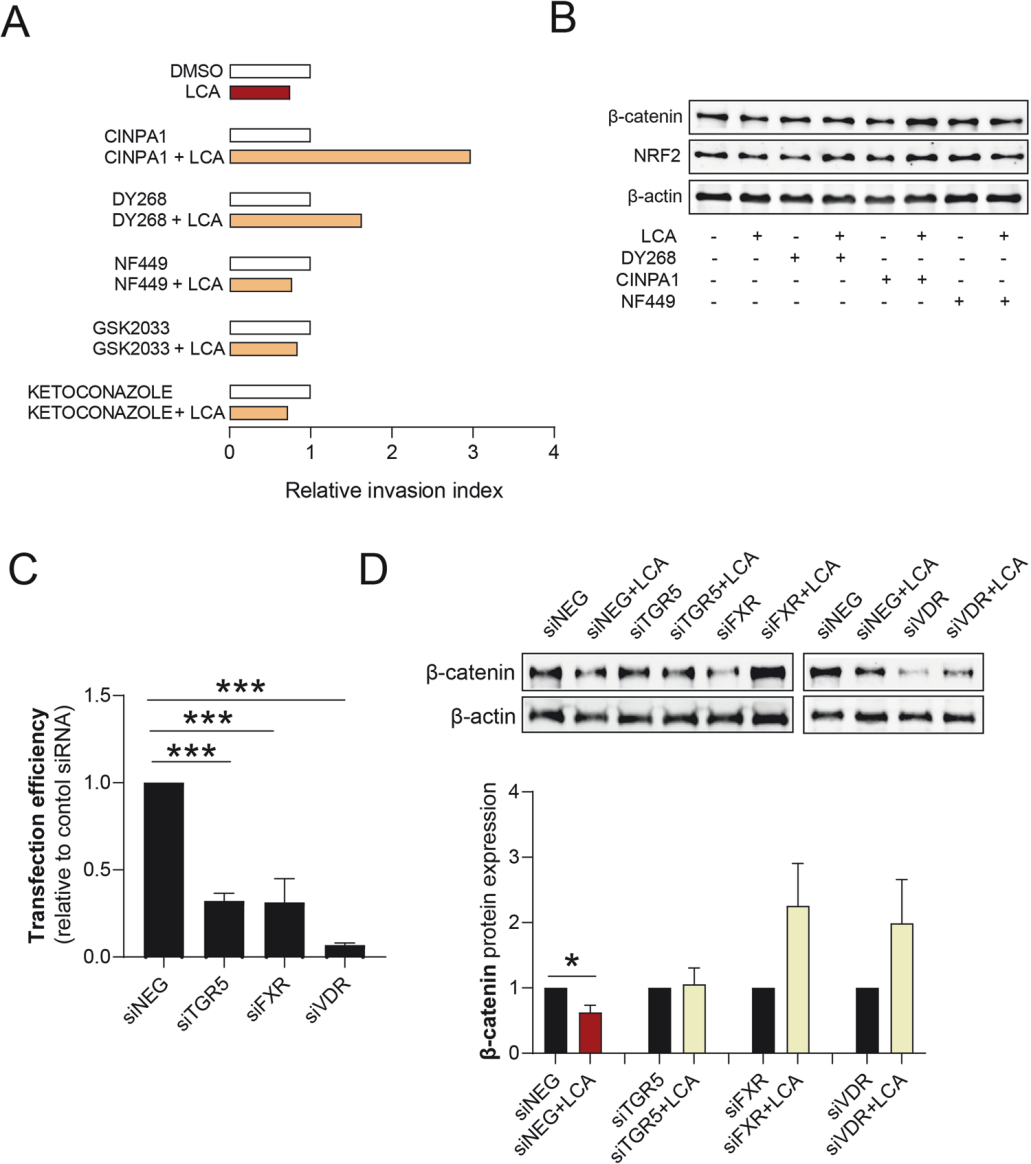
LCA-induced mesenchymal-epithelial transition (MET) and oxidative stress responses are mediated by the nuclear receptors, FXR, CAR, and VDR in pancreatic adenocarcinoma cells. Capan-2 cells were treated with DMSO or LCA (0.03 µM) and/or 5 µM CINPA1, DY268, NF449, GSK2033, and ketoconazole for 48 h. (**A**) Cell invasion was measured using Corning Matrigel invasion chambers. **B** β-catenin and NRF2 protein levels were measured by western blotting (representative figure). TGR5, FXR, and VDR bile acid receptors were transiently silenced in Capan-2 cells using the corresponding siRNA. A control group was transfected with negative control siRNA. After 48 h, **C** the transfection efficiency was assessed by qPCR, and **D** β-catenin expression was assessed by western blotting (*n* = 3). Data are presented as means ± SEM. Transfection efficiencies were compared with one-way ANOVA and Dunnett’s post hoc. Control siRNA vs. LCA/siRNA treated groups were compared with paired *t*-tests. *, **, and *** indicate *p* < 0.05, *p* < 0.01, and *p* < 0.001. DMSO dimethyl sulfoxide, FXR farnesoid X-activated receptor, LCA lithocholic acid, NRF2 nuclear factor, erythroid 2-like 2, TGR5 Takeda G-protein coupled receptor,VDR vitamin D receptor.

BAs can activate nuclear receptors, including farnesoid X receptor (FXR) [50, 51], constitutive androstane receptor (CAR) [9], pregnane X receptor (PXR) [52], liver X receptor (LXR) [53], and vitamin D receptor (VDR) [54], and membrane receptors, including G protein-coupled bile acid receptor 1 (GPBAR1, also known as TGR5) [7, 9, 25, 46, 55]. The receptors responsible for the effects of LCA in pancreatic adenocarcinoma cells were determined. First, LCA receptors were inhibited with antagonists and inhibitors, including CINPA1 to inhibit the CAR receptor, DY268 to inhibit the FXR receptor, NF449 to inhibit the downstream signaling pathway for the TGR5 receptor, GSK2033 to inhibit the LXR receptor, and ketoconazole to inhibit the PXR receptor. Reduced cell invasion in response to LCA treatment was blocked by CINPA1 and DY268; other receptor antagonists were ineffective (Fig. 7A). DY268 and CINPA1 blocked the suppression of β-catenin and NRF2 expression induced by LCA (Fig. 7B). For a more comprehensive view, we silenced the receptors using siRNAs in Capan-2 cells (Fig. 7C). VDR, another LCA receptor, was also assessed. Silencing of either FXR and VDR nuclear receptors blunted LCA-induced decreases in β-catenin protein expression. TGR5 receptor silencing did not affect LCA-induced changes (Fig. 7D). These results suggest that LCA effects are mediated by the CAR, FXR, and VDR receptors.

### LCA does not modulate the effectiveness of chemotherapy agents in pancreatic adenocarcinoma cells

**Fig. 8:**
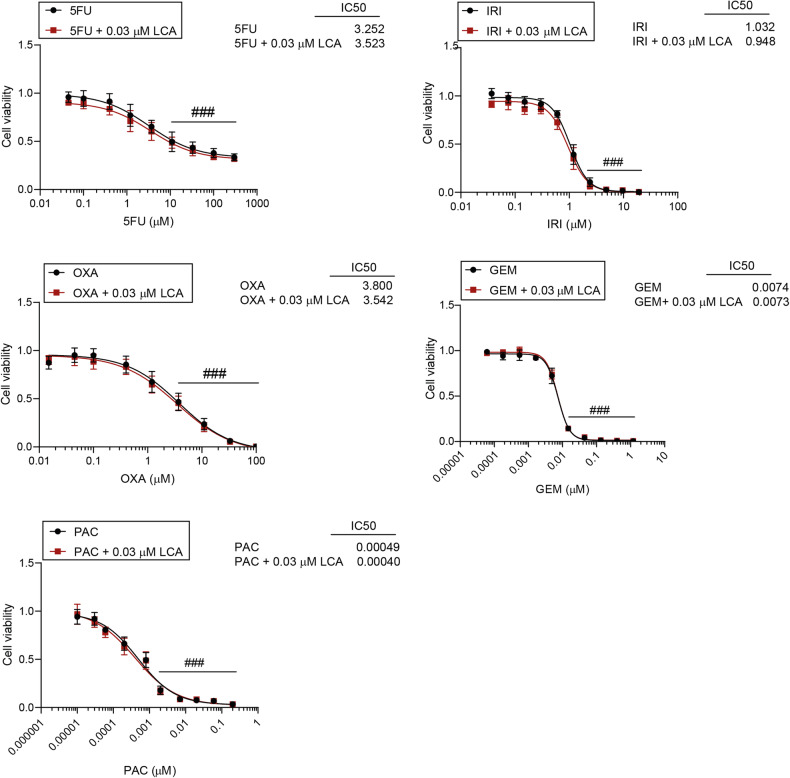
Assessment of the effects of LCA on the cytostatic activity of chemotherapy drugs. MTT assays were performed after 48 hr treatment of Capan-2 cells with chemotherapy compounds alone or in the presence of LCA (0.03 µM). Data are presented as means ± SEM from three biological replicates. Individual assays were measured in quadruplicate. Values were normalized to vehicle-treated cells. Nonlinear regression was performed on datasets to obtain IC_50_ values. Normality was determined using the D’Agostino and Pearson normality test. The 5-FU/LCA, GEM/LCA, OXA/LCA, and PAC/LCA datasets had normal distributions. IRI/LCA dataset normality was achieved by logarithmic transformation. Statistical differences were determined by two-way ANOVA, and all data points were compared with each other (in Tukey post hoc tests). #, ##, and ### indicate *p* < 0.05, *p* < 0.01, and *p* < 0.001, respectively, vehicle-treated vs. chemotherapy drug-treated cells. 5FU 5-fluorouracil, GEM gemcitabine, IRI irinotecan, LCA lithocholic acid, OXA oxaliplatin, PAC paclitaxel.

Next, the effects of LCA on the effectiveness of chemotherapy agents for the treatment of pancreatic adenocarcinoma were investigated. Gemcitabine, 5-fluorouracil, paclitaxel, oxaliplatin, and irinotecan were tested in different concentration ranges alone or in combination with LCA (0.03 µM) using MTT assays. IC_50_ values were determined using nonlinear regression analyses. We found that chemotherapy drugs were effective in pancreatic adenocarcinoma cells, but LCA did not enhance the effects of the drugs (Fig. 8).

## Discussion

LCA was identified as a bacterial metabolite with antineoplastic activity. Interestingly, bile acids were originally considered carcinogens [56, 57]. However, recent studies demonstrated that bile acids can be antineoplastic, and the pro- or anticarcinogenic activity of bile acids depends on the actual bile acid species in pancreatic adenocarcinoma (e.g. CDCA has procarcinogenic features [51], CA and UDCA have antineoplastic features [27, 58], and DCA has mixed effects [25, 51, 59]). LCA possesses antineoplastic features in neoplasias other than pancreatic adenocarcinoma, including breast cancer [7, 9, 46], neuroblastoma [40], prostate cancer [41, 42], liver cancer [45], gallbladder cancer [44], and nephroblastoma [43]. These effects are selective for neoplasias, as LCA did not affect primary, non-transformed cells [7]. Of note, the LCA concentrations in the present study were lower than the LCA concentrations in these studies.

The antineoplastic effects of LCA are multi-pronged, comprised of cytostasis, inhibition of EMT, reduction in cancer stem cell properties, and induction of mitochondrial oxidation. In terms of EMT, LCA suppressed mesenchymal marker (β-catenin and Snail) expression and increased epithelial marker expression (ZO1). In pancreatic cancer, increased expression of Claudin-1 is associated with disease progression [60, 61]. In our study, LCA treatment decreased the protein expression of Claudin-1 similar to mesenchymal markers. In line with the inhibition of EMT, LCA reduced invasion capacity and the cancer stemness programs in pancreatic adenocarcinoma cells. Of note, these programs are linked in many types of cancer, including pancreatic adenocarcinoma [62, 63].

Altered cellular metabolism is a hallmark of cancer [64] and extensive metabolic reprogramming is observed in pancreatic adenocarcinoma, including changes in glycolysis, mitochondrial oxidative phosphorylation and the Szentgyörgyi-Krebs cycle, lipid metabolism, and glutaminolysis. Although the underlying biochemical changes are diverse, one common feature is the suppression of mitochondrial OXPHOS [65, 66]. LCA treatment induced mitochondrial oxygen consumption, including fatty acid oxidation (etomoxir-sensitive respiration), glucose or amino acid oxidation (etomoxir-resistant respiration), and ATP production-linked respiration (oligomycin-sensitive respiration), but not uncoupled respiration (oligomycin-resistant respiration). Higher mitochondrial respiration levels may limit the availability of substrates for biosynthesis and contribute to metabolic inflexibility that renders cells less resilient to changes in nutrient availability.

Changes to the redox balance of cells were implicated as a major feature of the antineoplastic effects of LCA [9]. We showed that LCA induces oxidative and nitrosative stress in pancreatic adenocarcinoma cells through suppressing NRF2 and inducing iNOS, which plays a key role in suppressing EMT and, in cell models of other neoplasias, proliferation [9]. The carcinogenic effects of NRF2 in pancreatic cancer have been demonstrated in several studies [67–69]. In agreement with this, ROS overproduction renders pancreatic adenocarcinoma cells susceptible to cell death [70], and the progression of pancreatic adenocarcinoma often coincides with mutations inactivating Keap-1 and rendering NRF2 constitutively active [71]. High nuclear NRF2 expression correlates with reduced pancreatic cancer patient survival rates [72]. We provide evidence that antioxidants are overexpressed in pancreatic adenocarcinoma and that overexpression is associated with worse clinical outcomes.

In this study, LCA exerted antineoplastic effects through CAR, FXR, and VDR nuclear receptors in cell models of pancreatic adenocarcinoma. VDR is highly expressed in pancreatic cancer cells [73] and pancreatic tumor stroma [74]. The activation of VDR signaling enhances pancreatic cancer therapy [74, 75]. VDR receptor has also been implicated in suppressing pancreatic cancer cell stemness [76]. Understanding the effects of FXR is more difficult, as studies concerning the effects of high FXR expression on cell survival are contradictory [77, 78]. The effects of the CAR receptor in pancreatic adenocarcinoma are not known. BAs can activate a plethora of receptors (reviewed in [30]) and similar pathways may play a role in inducing LCA-mediated antineoplastic programs in other cancers (e.g. colon cancer [54]), but different receptors may be activated in other neoplasias to elicit cytostasis (e.g. TGR5 and CAR receptors in breast cancer [9]).

Microbial metabolites also play an important role in pancreatic adenocarcinoma responses to therapy [79–81]. To the best of our knowledge, the effects of LCA on chemo-, radiation, and targeted therapy in PDAC are not known. Bile acids can be advantageously combined with anticancer agents. In chemotherapy-treated pediatric patients, increased circulating BA levels correlate with faster recovery. TUDCA supplementation resulted in better recovery after 5-FU treatment through inhibition of ER stress responses in mice [82]. UDCA enhanced DNA topoisomerase I inhibitor-induced apoptosis in several cancer cell lines [83]. Combining UDCA with the COX-2 inhibitor, celecoxib, reduced colon cancer cell growth [84]. Interestingly, UDCA has synergistic effects on the antitumor activity of sorafenib in hepatocellular carcinoma cells [85]. In our study, the combination of LCA and chemotherapy drugs used in the treatment of pancreatic adenocarcinoma did not affect cancer cell growth.

While the experimental results equivocally demonstrate that LCA can block multiple cancer hallmarks in cell models of pancreatic adenocarcinoma, whether these effects are translated into human pancreatic adenocarcinoma is unclear. Bile acids are associated with risk factors of pancreatic adenocarcinoma, such as obesity, diabetes, pancreatitis, and hypertriglyceridemia. For example, the concentration of DCA is elevated in type 2 diabetes [86], *ob/ob* mice have elevated plasma bile acid levels [87], and circulating total bile acid levels are increased in pancreatitis [88].

Conjugated bile acid levels (glycocholic acid detected in most cases) are elevated in the plasma and serum samples from PDAC patients compared to controls [51, 89–93]. The levels of unconjugated bile acids in the plasma of PDAC patients are reduced [92]. However, the concentration of unconjugated bile acids in bile from the common bile duct (CBD) increased in PDAC patients compared to patients with benign disease. The increased unconjugated bile acid concentration may be due to hydroxylase-producing bacteria in the bile duct and CBD stones that block bile flow, leading to bile stasis and bacterial overgrowth [94]. Bile acid concentrations in human serum may help distinguish patients with pancreatic adenocarcinoma from patients with benign diseases and healthy individuals.

To the best of our knowledge, no information about the association between bacterial LCA production in pancreatic cancer and cancer progression has been published. Of note, the level of LCA is the lowest among secondary bile acids in the serum of healthy individuals [30]; thus, LCA can be difficult to detect. In other cancers, such as breast adenocarcinoma, bacterial LCA conversion is reduced at an early stage (stage 0–1), suggesting that low LCA levels contribute to the pathogenesis of breast cancer [7].

## Conclusions

Hereby we demonstrate that LCA induces antineoplastic features in pancreatic adenocarcinoma cells. LCA inhibits the proliferation of pancreatic adenocarcinoma cells, EMT, and cancer stem cell marker expression and induces mitochondrial oxidative phosphorylation. LCA induces oxidative/nitrosative stress, which is the root of the antineoplastic features of LCA. These effects are mediated through CAR, FXR, and VDR nuclear receptors. Our studies highlight LCA as a non-toxic antineoplastic compound and point out the pharmacological exploitability of the nuclear receptors responding to LCA.

## Materials and methods

### Chemicals

LCA (cat # L6250; Sigma-Aldrich, St. Louis, MI, USA) was dissolved in DMSO at a stock concentration of 100 mM. LCA was used at a concentration of 0.03 µM, corresponding to normal human serum concentration [7, 47, 48]. Non-treated cells received 0.001% DMSO in the medium as a vehicle. Glutathione (GSH; cat # G4251; Sigma-Aldrich) was used at a final concentration of 5 mM. Pegylated catalase (pegCAT; cat # C4963; Sigma-Aldrich) was used at a concentration of 500 U/ml. BA receptor antagonists (NF449 [95], CINPA1 [96], DY268 [97], and GSK2033 [98]) were acquired from Tocris Bioscience (Bristol, UK), and ketoconazole [99] was purchased from Sigma-Aldrich. NF449 (G_sα_-selective antagonist; 5 µM) was used to inhibit TGR5 signaling. Nuclear receptor activation was inhibited using 5 µM CINPA1 (CAR antagonist), DY268 (FXR antagonist), and GSK2033 (LXR antagonist). PXR downstream signaling was inhibited using ketoconazole (5 μM). Chemotherapy drugs (irinotecan, paclitaxel, gemcitabine, 5-fluorouracil and oxaliplatin) were purchased from Sigma-Aldrich. Silencer Select siRNAs targeting TGR5 (GPBAR1-siRNA ID: s195791), VDR (NR1I1- siRNA ID: s14777), and FXR (NR1H4-siRNA ID: s19371), and the negative control siRNA #1 (cat # 4390843) were purchased from Thermo Fisher Scientific (Waltham, MA, USA) and used at a final concentration of 30 nM.

### Cell lines

Capan-2 and BxPC-3 human pancreatic adenocarcinoma cell lines were purchased from the American Type Culture Collection. Capan-2 cells were grown in MEM (Sigma-Aldrich, cat # M8042) supplemented with 10% fetal bovine serum (FBS), 1% penicillin/streptomycin, and 2 mM glutamine. BxPC-3 cells were cultured in RPMI 1640 (Sigma-Aldrich; cat # R5886) containing 10% FBS, 1% penicillin/streptomycin, and 2 mM glutamine. All cells were cultured at 37 °C in a humidified incubator with 5% CO_2_. Cell lines were regularly monitored for mycoplasma contamination.

### Cell proliferation assays

Cells were added to 96-well plates (3000 cells/well) in 200 µl of complete medium. After treatment with LCA or vehicle (DMSO) for 48 h, cell proliferation was assessed using MTT and SRB assays. For MTT assays, cells were treated with MTT solution (20 µl of 5 mg/ml) and incubated at 37 °C for 1.5 h. After discarding the supernatants, the formazan crystals were solubilized in 100 µl DMSO/well, and absorbance at 540 nm was measured on a plate reader (Thermo Labsystems Multiskan MS, Walthman, MA, USA). For the SRB assay, cells were fixed with trichloroacetic acid (TCA, 10% final concentration) for 1 h at 4 °C. After washing with water, the cells were stained with SRB solution (0.4% in 1% acetic acid). The unbound dye was removed with 1% acetic acid. The bound stain was dissolved in 10 mM Tris base and the absorbance was measured at 540 nm.

### Cell invasion assay

Cell invasion was assessed utilizing Corning BioCoat Matrigel Invasion Chambers (Corning, NY, USA), as described by Schwarcz et al. [59].

### Western blotting

RIPA buffer (50 mM Tris, 150 mM NaCl, 0.1% SDS, 1% TritonX 100, 0.5% sodium deoxycholate, 1 mM EDTA, 1 mM Na_3_VO_4_, 1 mM NaF, 1 mM PMSF, and protease inhibitor cocktail) was used for cell lysis. Protein concentrations were measured using a BCA protein assay kit (Pierce Biotechnologies, Rockford, IL, USA). Proteins (20 µg) were separated by electrophoresis (10% SDS polyacrylamide gel) and transferred to nitrocellulose membranes. After blocking in 5% BSA for 1 h at room temperature, membranes were incubated with primary antibodies (Table 1) at 4 °C overnight. After washing, membranes were incubated with IgG HRP-conjugated secondary antibody (Cell Signaling Technology, Inc. Beverly, MA, 1:2000) for 1 h. Antibody binding was detected with chemiluminescence (SuperSignal West Pico Solutions, Thermo Fisher Scientific). β-actin was used as a loading control. Blots were quantified by densitometry using Image Lab 6.1 software.

**Table 1:** Primary antibodies for Western blotting.

Antibody symbol	Vendor	Dilution
β-catenin	Cell Signaling Technology (8480)	1:1000
Snail	Cell Signaling Technology (3879)	1:1000
Claudin-1	Cell Signaling Technology (13255)	1:1000
ZO1	Cell Signaling Technology (8193)	1:1000
NRF2	Abcam (ab31163)	1:1000
iNOS	Novus (NB300-605)	1:1000
4HNE	Abcam (ab46545)	1:1000
Nitrotyrosine	Thermo Fisher Scientific (A21285)	1:1000
ALDH1	Abcam (ab227948)	1:1000
CD133	Novus (NB120-16518SS)	1:1000
β-actin	Sigma-Aldrich (A3854)	1:20000

### Aldefluor assay

The Aldefluor assay (Aldefluor Stem Cell kit; StemCell Technologies, Vancouver, Canada) was performed to identify aldehyde dehydrogenase (ALDH) positive cells as cancer stem-like cells similar to [59].

### Quantitative RT-PCR

RNA was isolated with TRIzol reagent (Invitrogen, Waltham, MA, USA), and the corresponding cDNA was generated with a High Capacity cDNA Reverse Transcription Kit (Applied Biosystems, Waltham, MA, USA). The qPCR reaction consisted of 500 nM primers (Table 2) and qPCRBIO SyGreen Lo-ROX Supermix (PCR Biosystems Ltd., London, UK). The mRNA levels were normalized to the geometric mean of 36B4 and cyclophilin (CYCLO) expression.

**Table 2:** Primers for RT-qPCR.

Gene	Forward primer (5’–3’)	Reverse primer (5’–3’)
*TGR5*	CACTGTTGTCCCTCCTCTCC	ACACTGCTTTGGCTGCTTG
*FXR*	TGCTTACAGCAATTGTTATCCTG	ACATCAAGAAGTGGCTCCTGA
*VDR*	GGACTGCCGCATCACCAA	TCATCTCCCGCTTCCTCT
*36B4*	CCATTGAAATCCTGAGTGATGTG	GTCGAACACCTGCTGGATGAC
*CYCLO*	GTCTCCTTTGAGCTGTTTGCAGAC	CTTGCCACCAGTGCCATTATG

### Oxygen consumption and extracellular acidification rate

The oxygen consumption rate (OCR) and extracellular acidification rate (ECAR) were measured in LCA-treated cells using a Seahorse XF96 analyzer (Agilent Technologies, Santa Clara, CA, USA). Cells were allowed to attach overnight after seeding in 96-well Seahorse assay plates (5000 cells/well). After treating with vehicle (DMSO) or LCA for 48 h, cells were incubated in pre-warmed XF assay media for 1 h at 37 °C in a non-CO_2_ incubator. Baseline OCR was recorded 5 times for 5 min. Then, cells were treated sequentially with etomoxir (50 μM), oligomycin (10 μM), and antimycin (10 μM). Each OCR was recorded 5 times for 5 min. All measurements were normalized to protein levels using the SRB assay. LCA treatment was represented in 6-8 wells per experiment and replicate experiments were performed three times. Fold change values were calculated.

Basal respiration was defined as the baseline respiration minus the antimycin-resistant respiration. Etomoxir-resistant OCR (etomoxir−antimycin) was defined as the oxygen consumption related to glucose and amino acid oxidation. Fatty acid oxidation was defined as etomoxir-sensitive OCR (baseline−etomoxir). Uncoupled respiration was defined as oligomycin-resistant respiration (oligomycin−antimycin), and ATP-linked respiration was defined as oligomycin-sensitive OCR (baseline−oligomycin).

### Transfection with siRNA

For transient transfection, Capan-2 cells were treated with 30 nM TGR5, VDR, FXR siRNA or negative control siRNA. Transfection was performed using Lipofectamine RNAiMAX. Transfection was performed for 2 days with and without LCA.

### Statistical analyses

Experiments were independently repeated at least three times. The results are presented as the means ± SEM. Normality was determined using the D’Agostino–Pearson normality test. For comparison of control and LCA-treated groups paired t-test was used. One- or two-way analysis of variance test followed by Dunnett’s or Tukey’s honestly significant post hoc test were used for multiple comparisons. The “[Inhibitor] vs. response-variable slope (four parameters)” utility was used to perform nonlinear regression for the determination of IC_50_ values. Statistical analyses were performed using GraphPad Prism 8 software.

### Supplementary information

Original Data

## Data Availability

Primary data is available at https://figshare.com/s/683de18ac1c05f3e4762 (10.6084/m9.figshare.24087399).
