# Supplementary figures and images for: The bacterial metabolite, lithocholic acid, has antineoplastic effects in pancreatic adenocarcinoma
Source: Cell Death Discov. 2024 May 23;10:248. doi: 10.1038/s41420-024-02023-1 (PMC11116504; doi:10.1038/s41420-024-02023-1)

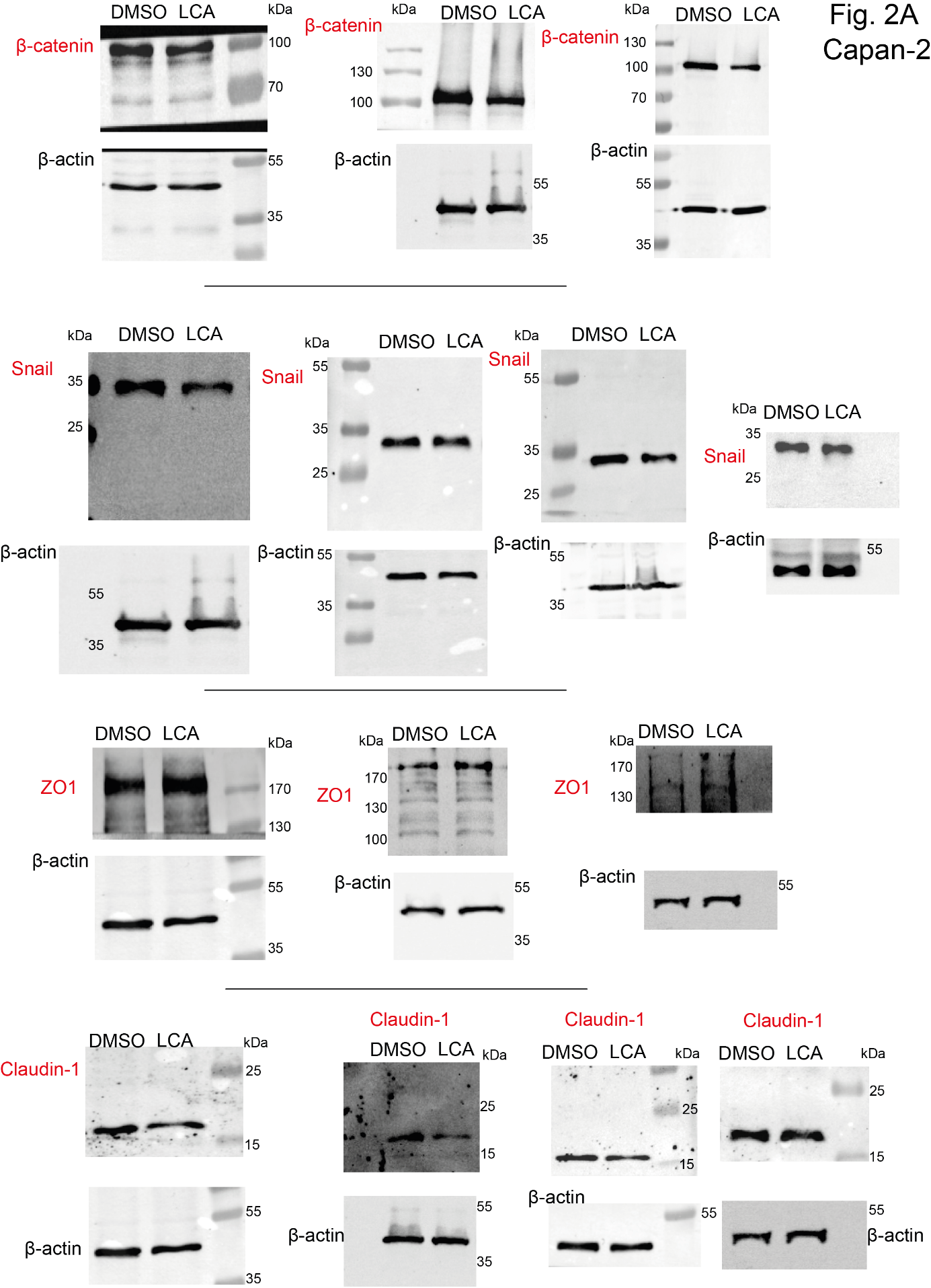


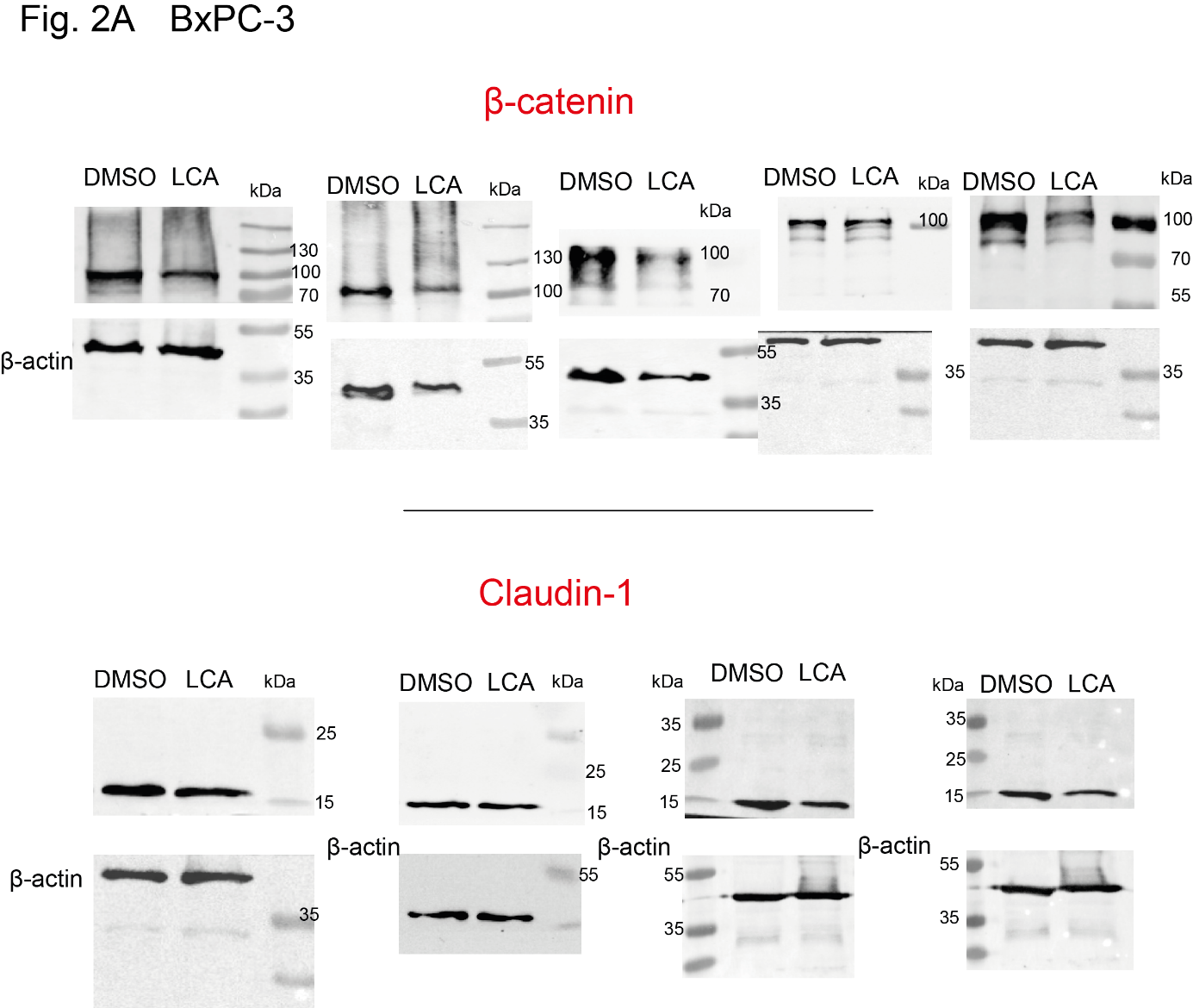


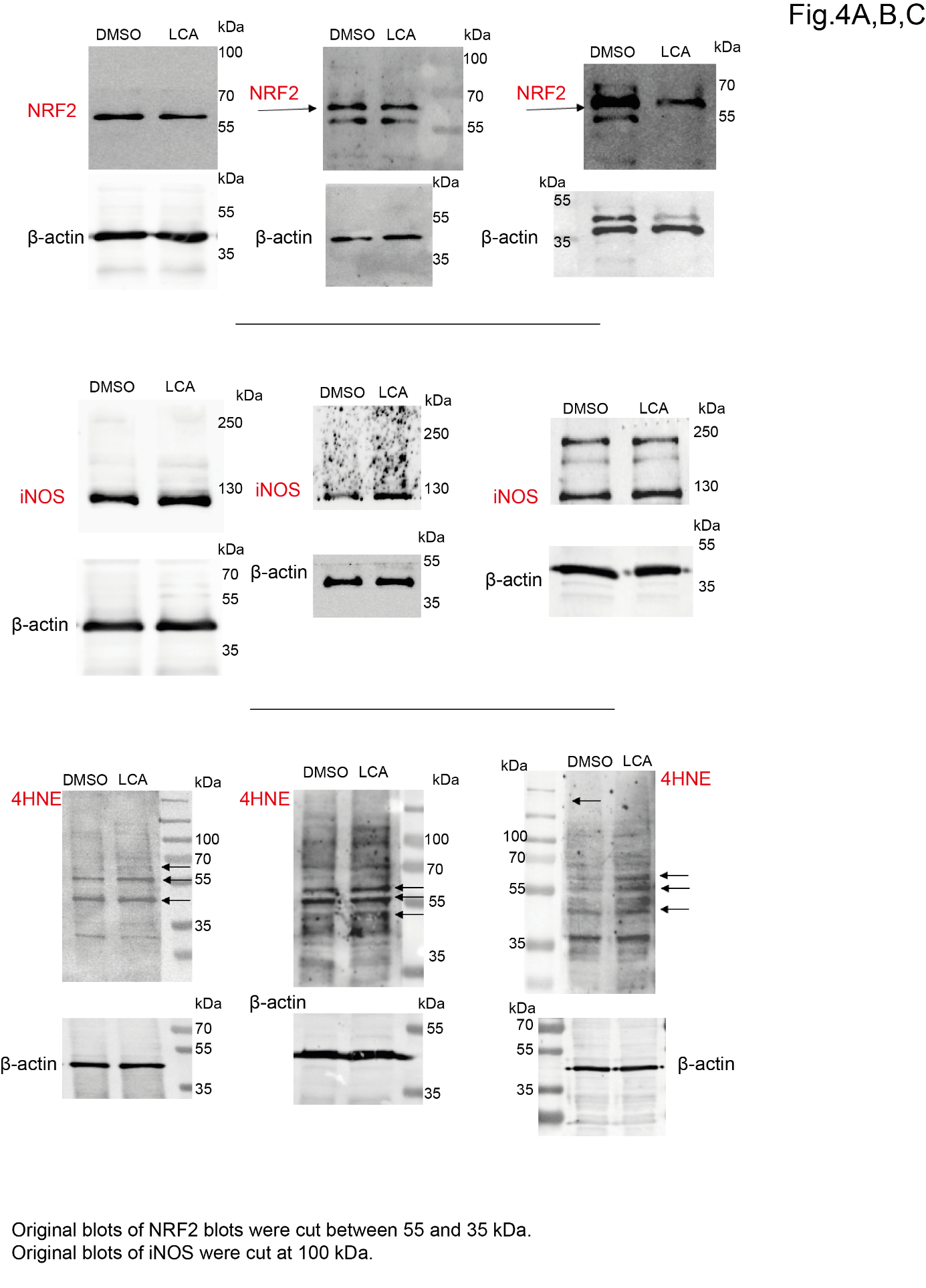


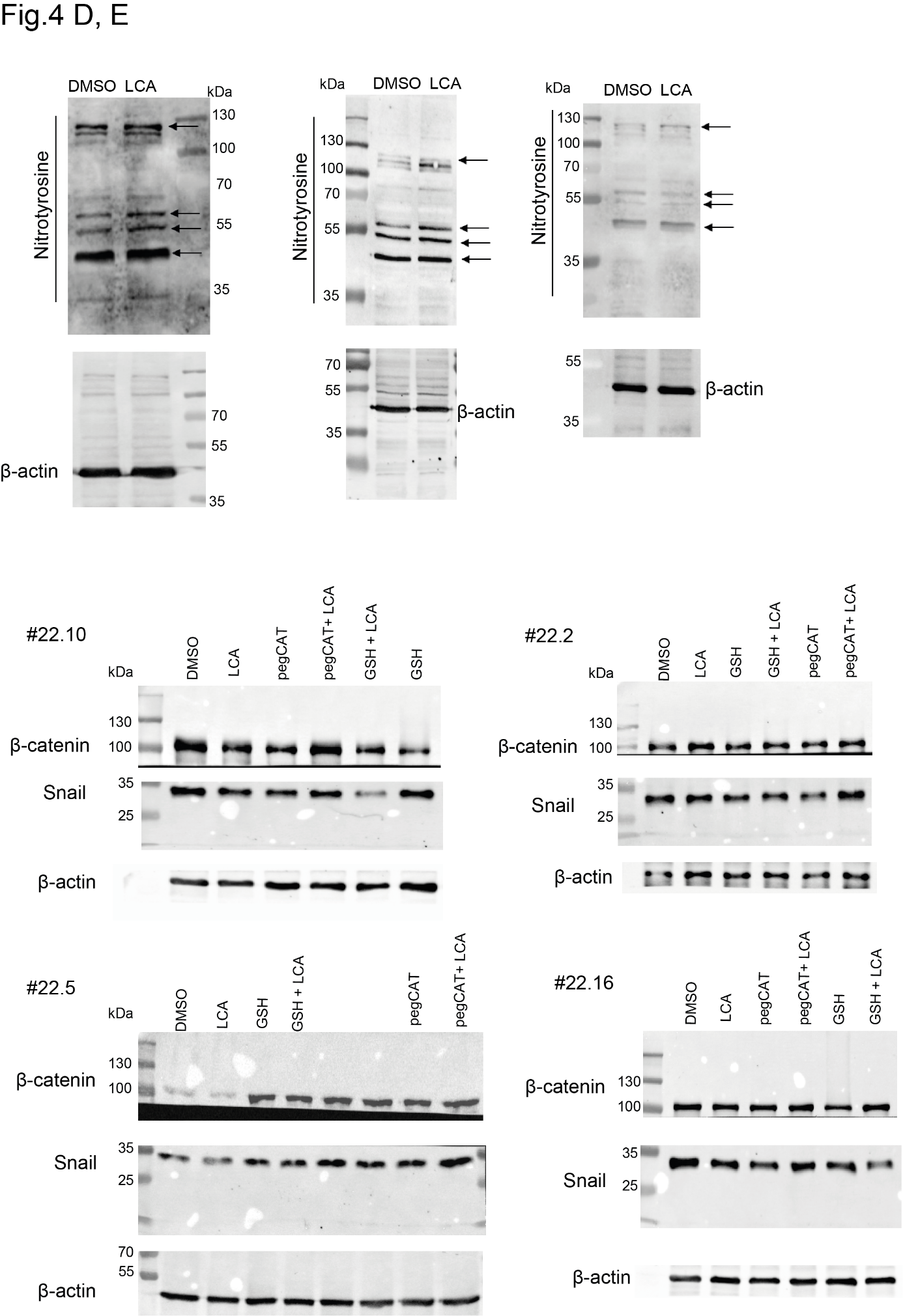


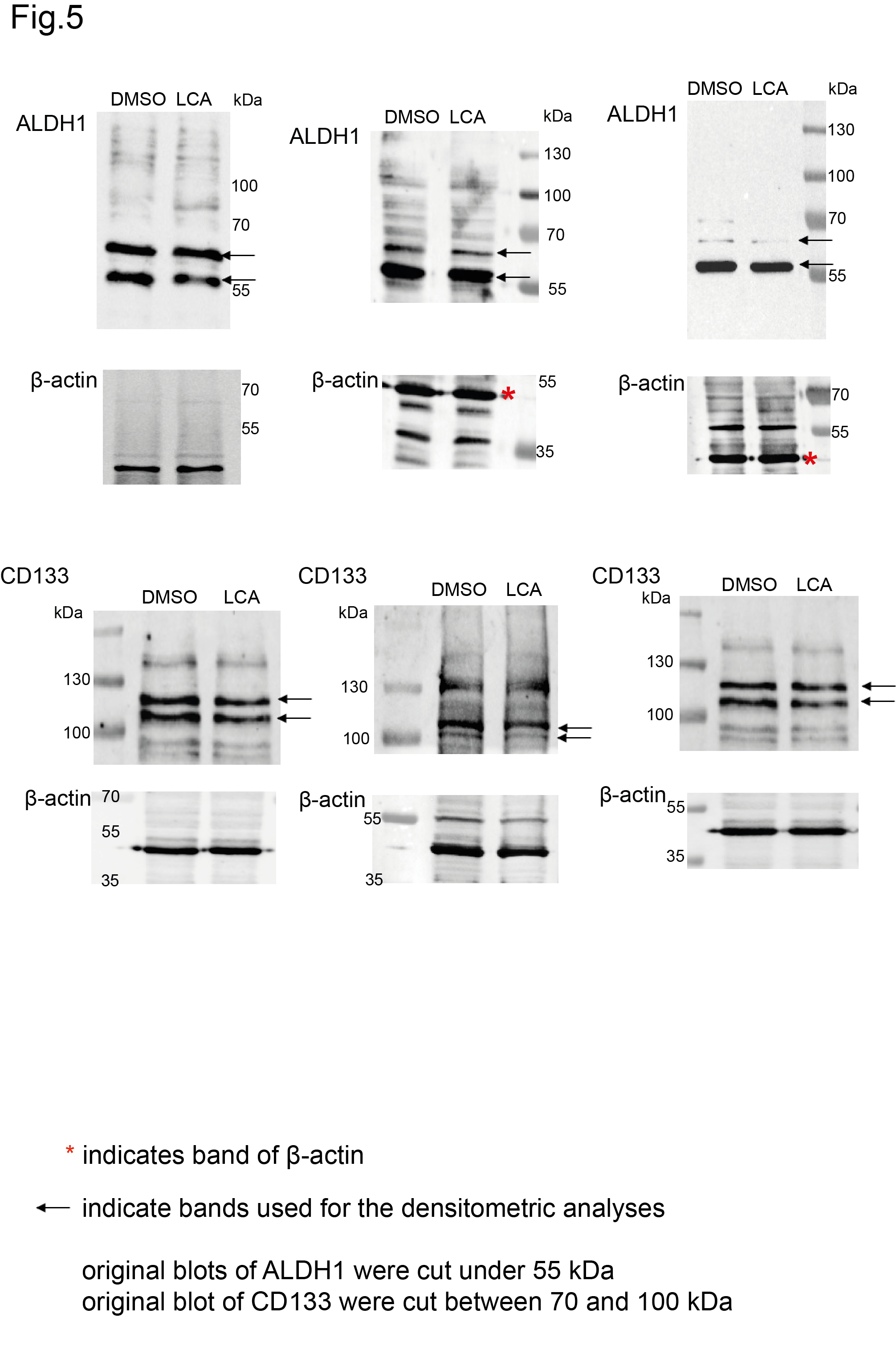


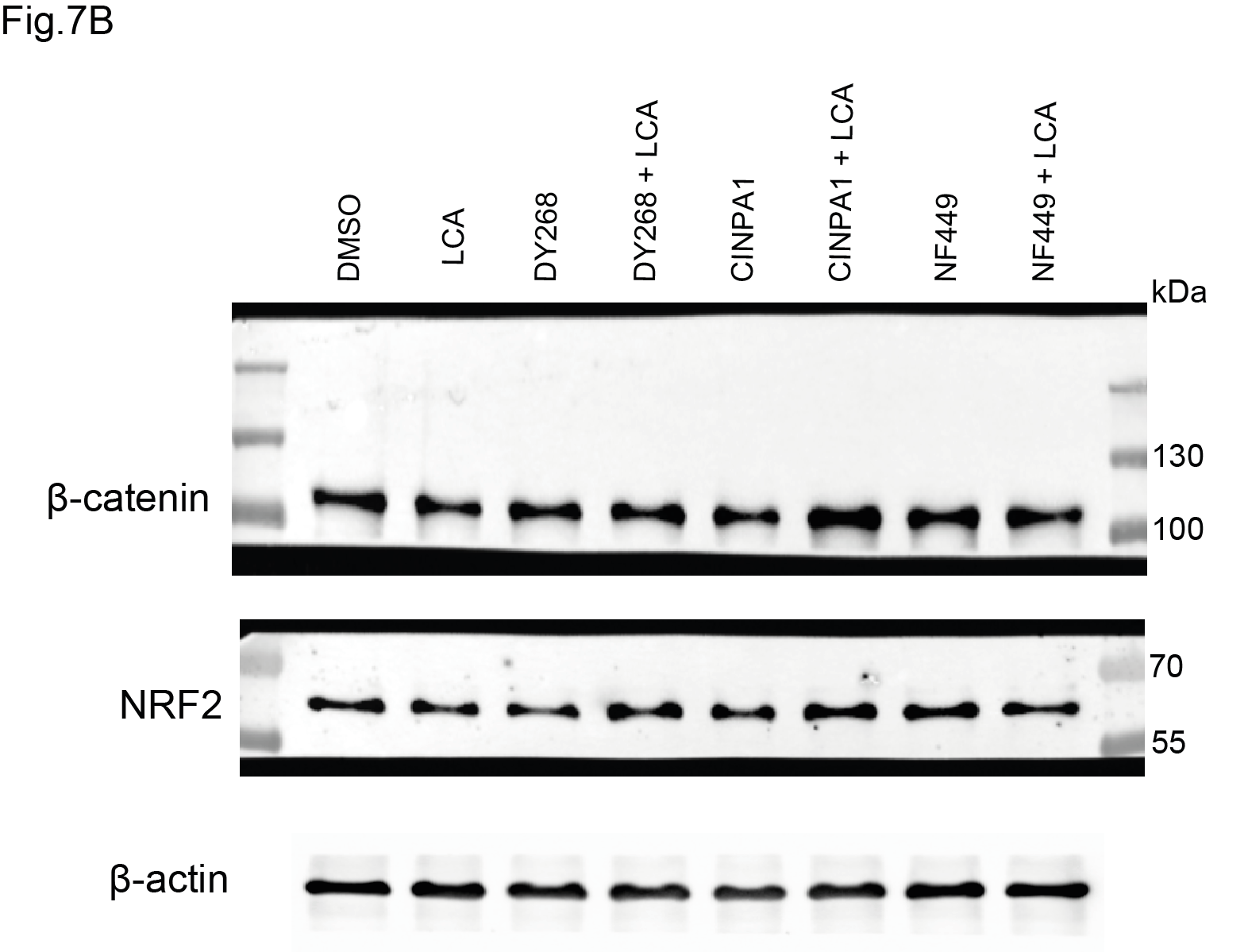


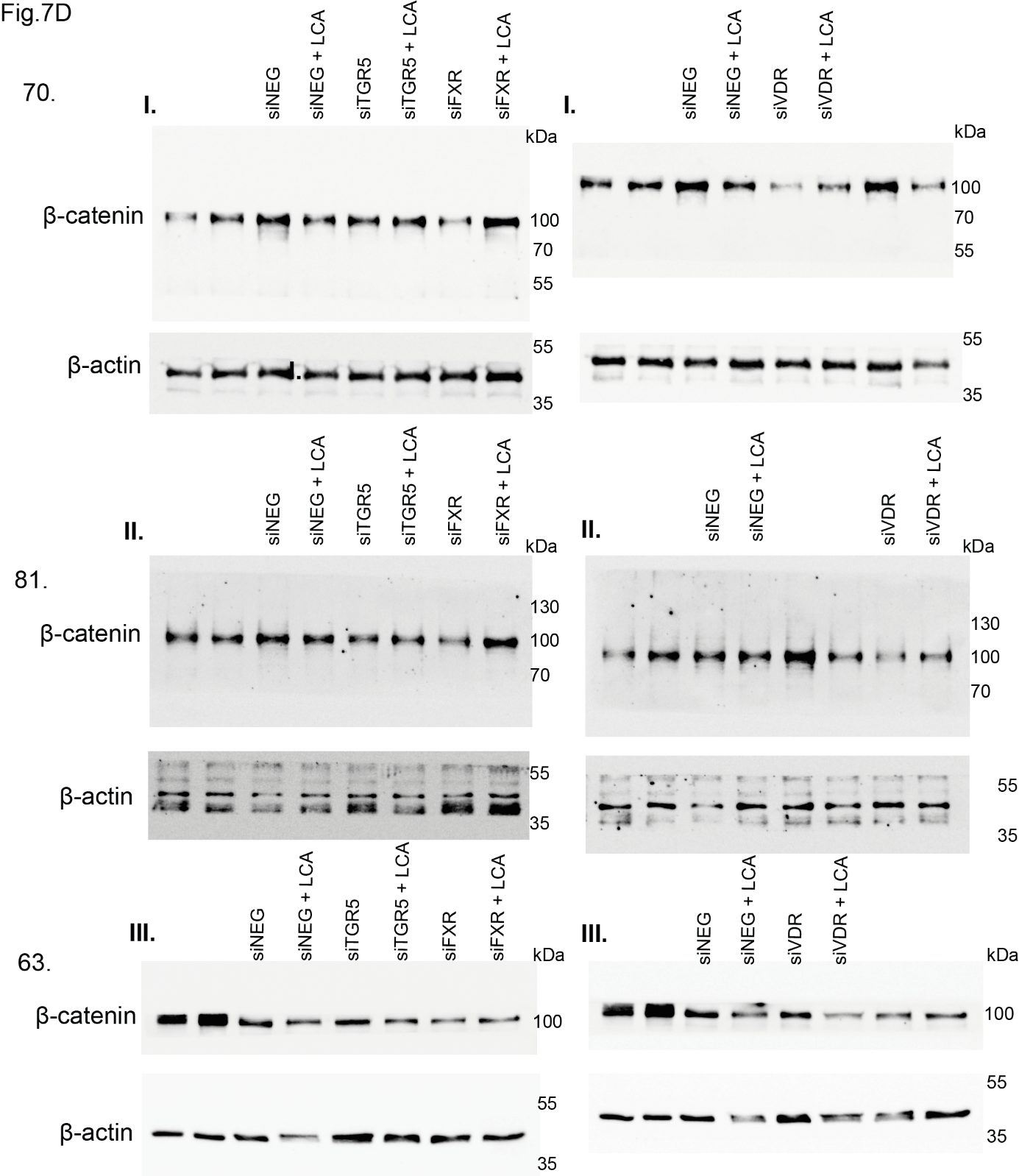

Supplement: Supplementary file 1 — Original Data [file 41420_2024_2023_MOESM1_ESM.docx]
